# Principles of Industry-Academic Partnerships Informed by Digital Mental Health Collaboration: Mixed Methods Study

**DOI:** 10.2196/77439

**Published:** 2025-09-10

**Authors:** Sophie S Hall, Olivia Hastings, Kelly Marie Prentice, Beverley Brown, Jacob Andrews, Sonal Marner, Rebecca Woodcock, Jennifer Martin, Charlotte L Hall

**Affiliations:** 1 Nottingham Clinical Trials Unit, School of Medicine, University of Nottingham, University Park Campus, Nottingham, NG7 2UH, United Kingdom, 44 7817139077; 2 National Institute of Health and Care Research MindTech HealthTech Research Centre, School of Medicine, University of Nottingham, Nottingham, United Kingdom; 3 Public and Patient Contributor, National Institute of Health and Care Research MindTech HealthTech Research Centre, School of Medicine, University of Nottingham, Nottingham, United Kingdom; 4 National Institute for Health and Care Research Nottingham Biomedical Research Centre, Institute of Mental Health, University of Nottingham, Nottingham, United Kingdom; 5 Mental Health and Clinical Neurosciences, School of Medicine, University of Nottingham, Queen’s Medical Centre, Nottingham, United Kingdom

**Keywords:** industry-academic partnerships, mental health, innovation, challenges, guidance, collaboration

## Abstract

**Background:**

Cross-sector collaboration is increasingly recognized as essential for addressing complex health challenges, including those in mental health. Industry-academic partnerships play a vital role in advancing research and developing health solutions, yet differing priorities and perspectives can make collaboration complex.

**Objective:**

This study aimed to identify key principles to support effective industry-academic partnerships, from the perspective of industry partners, and develop this into actionable guidance, which can be applied across sectors. Mental health served as a motivating example due to its urgent public health relevance and the growing role of digital innovation.

**Methods:**

Using a 3-stage, mixed-methods approach, we conducted a web-based survey of UK-based digital mental health companies (N=22) to identify key barriers and facilitators to industry-academic partnerships. This was followed by 2 focus groups (n=5) that explored emerging themes from the survey using thematic analysis. Finally, we conducted a workshop with industry representatives, researchers, clinicians, and PPI members to co-develop the Principles of Industry-Academic Partnerships (PIP) guidance.

**Results:**

Survey findings highlighted that industry partners valued academic collaboration for enhancing credibility, facilitating knowledge transfer, and gaining access to PPI networks. However, key barriers included high costs, slow academic timelines, and complex contracting processes. The 4 major themes that emerged from the focus groups were: advantages of collaboration, cultural differences between organizations, collaboration models, and structural barriers within universities. Through informed discussions in the workshop, these themes were explored, leading to the development of 14 actionable strategies. These strategies reflect industry perspectives and formed the PIP guidance, categorized under project initiation, defining the scope and agreements, project execution, and promoting sustainability.

**Conclusions:**

The PIP guidance provides a practical framework to support more effective and mutually beneficial collaborations between industry and academia. Developed through the lens of mental health research, the strategies identified are broadly applicable across disciplines where cross-sector partnerships are essential. Industry partners valued academic collaborations for their credibility and scientific rigor, but highlighted persistent structural and cultural barriers within universities. Addressing these challenges by aligning expectations and timelines, adopting flexible collaboration models, and streamlining operational processes can help foster impactful and sustainable partnerships in mental health and beyond.

## Introduction

Mental health disorders constitute a global public health challenge, with growing demands for more effective treatments, interventions, and policy solutions [1,2]. The integration of expertise, resources, and diverse perspectives from both academic institutions and industry partners is increasingly essential to address the complex biological, psychological, and societal dimensions of mental health [3]. While here we focus on digital mental health as a motivating case example, the challenges and opportunities identified are broadly relevant to other sectors where interdisciplinary and cross-sector collaboration is critical. Despite the potential benefits, forming successful and sustainable industry and academic partnerships remains a challenge. Between 2016 and 2017 and between 2018 and 2019, the National Health Service (NHS) in the United Kingdom received £9000 ($12,156) per patient enrolled in commercial trials and saved £5800 ($7834) per patient in drug costs, generating £355 million ($471,520) in income and £28.6 million ($38,632) in savings [4]. However, patient recruitment to industry-funded research has halved over the past 5 years, costing the NHS around £360 million ($486,274) [5].

In this study, we refer to the “industry sector” as organizations involved in the development, commercialization, and delivery of digital mental health technologies, including start-ups, small to medium enterprises (SMEs), and larger corporations. The “academic sector” refers to university-based researchers and research institutions engaged in the evaluation, design, and theoretical advancement of mental health interventions. While these roles can sometimes overlap, particularly in collaborative or spin-out models, we use these definitions to distinguish the differing priorities, structures, and operational cultures that shape partnership dynamics. Improving industry and academic partnerships in mental health research could accelerate the development of new treatments, digital health tools, and public health strategies to help address the United Kingdom’s mental health crisis [3]. Recent advances in artificial intelligence, big data analytics, and precision medicine present an opportunity for new forms of partnership that can leverage the industry’s technological capabilities and access to resources with academia’s research expertise. However, across areas of study (ie, not specific to the mental health field), academia and industry have traditionally been viewed as having different and often contrasting priorities [6,7]. Traditionally, academia has played a central role in advancing foundational knowledge in mental health, focusing on epidemiology, neuroscience, and therapeutic approaches. Academic researchers often prioritize long-term studies, driven by a commitment to high-quality scientific inquiry, resulting in high-impact outputs (eg, academic publications) with an emphasis on data sharing and transparency. Industry, on the other hand, excels in translating research into practical applications, with a focus on commercialization, rapid innovation, and the scalability of interventions. Industry partners are often concerned with protecting intellectual property. These differences can lead to mistrust and misaligned priorities or reluctance from either side to engage fully, which can hinder the development of impactful, patient-centered mental health solutions [6,8-10].

Despite growing interest in exploring the motivations, barriers, and facilitators to industry-academic partnerships [6,8-10], there is a lack of UK-based research and a lack of research specific to mental health collaborations. Further investigation is needed to understand the specific challenges facing the mental health field, and a solution-focused approach is required to develop actionable insights. A recent paper identified that clear guidance for industry-academic collaborations in the field of mental health is lacking, but may support successful collaborative practice [11]. Although the authors of this paper noted that such guidance may include the importance of supporting shared values around a commitment to ethical principles and patient and public involvement (PPI), as well as data sharing, specific details, and strategies were not provided. There is a time-critical need for collaborations to be better developed to ensure transparency in partnerships’ accessibility of data and to support independent verification of results [11]. Indeed, there are calls to action to improve awareness of each partner’s needs and the barriers and facilitators to successful partnerships [12-14].

This study aimed to identify key principles to support effective industry-academic partnerships from the perspective of industry partners and develop actionable guidance to support such partnerships. With the aim of developing this early-stage guidance, we conducted a web-based survey and focus groups to explore common barriers and facilitators and hosted a workshop to translate the learning from these into actionable guidance statements. Due to the pressing societal relevance and growing role of digital innovation, mental health served as the motivating context to develop this guidance. However, the study did not focus on any single diagnostic category. Instead, it examined collaboration across the broader digital mental health sector, which could also be applied more broadly to other health sectors. The resulting guidance addresses cross-cutting structural and relational factors that influence collaboration, offering insights that are applicable across a range of disciplines where interdisciplinary and cross-sector partnerships are critical.

## Methods

### Patient and Public Involvement

PPI was embedded throughout the project and reported according to the GRIPP2-SF (Guidance for Reporting Involvement of Patients and the Public-Short Form) [15]. The aim of including PPI was to ensure that the guidance was not just shaped by institutional or commercial priorities but was anchored in the real-world needs, values, and concerns. Two PPI members, with lived experience of participating in mental health research with industry partners, contributed throughout the study. They participated in iterative piloting of the survey before launch (Stage 1), informed the development of the focus group topic guide through discussions and interpretation of survey findings (Stage 2), and were invited to an online workshop to collaboratively review and agree on the key statements forming the final guidance (Stage 3).

### Stage 1: Survey

#### Recruitment and Participants

We aimed to recruit 20‐25 participants to a web-based survey to provide an initial understanding of the benefits and challenges that industry partners experience when considering evaluating their product through an academic partner research study or trial. Participants were recruited to the survey through targeted email invitations to UK commercial mental health companies, identified through the study teams' contacts, through internet searches, and through disseminating study adverts at the Giant Health London 2024 conference. All participants who completed the consent form completed the survey. Inclusion criteria included:

1. Industry professional: individuals who were currently employed or contracted in a professional capacity within the mental health, technology, or health care sectors and not via an academic institution.
2. Working in digital mental health: participants must have been actively involved in work related to digital mental health, defined as the use of digital tools, platforms, or services aimed at supporting mental health care, prevention, or well-being. This includes work on apps, web-based therapy platforms, digital interventions, AI-based mental health tools, or related infrastructure.
3. Have a UK base: participants must be based in the United Kingdom, either through their primary place of work or residence, to ensure relevance to the UK digital mental health landscape.

There were no exclusion criteria.

#### Survey Design

The survey consisted of a mix of closed (eg, Likert scale) response and open-ended (eg, free text box) style questions. The survey was an “open” web-based survey hosted on Research Electronic Data Capture (REDCap; Vanderbilt University Medical Center), a secure web application for building and managing web-based surveys and databases and open from November 12, 2024, to January 9, 2025. The survey was iteratively piloted through multiple rounds of feedback and revision before its launch. No changes were made after being launched. The survey was conducted according to the CHERRIES (Checklist for Reporting Results of Internet E-Surveys) checklist [16] (see Appendix B in Multimedia Appendix 1). Cookies or intellectual property (IP) checks were not used to assign unique identifiers. Participants’ email addresses were checked to assess duplication (there was no duplication).

#### Instruments

A survey comprised 30‐37 items (depending on whether they had or had not collaborated with academics previously), including 10 background questions relating to their organizations, 2 questions on collaboration with health care professionals, 3 questions on working with PPI, 15 questions on their experiences collaborating with academics, or alternatively 22 questions if they had not previously worked with academics. Items are presented in Appendix C in Multimedia Appendix 1, but typically asked participants to rate how strongly they agreed with negative and positively worded statements. All items had to be completed to allow submission of the survey.

#### Procedure

The study advert provided with a brief description alongside a website link and QR code, which linked to the survey REDCap database. Participants who clicked on this link were directed to the participant information sheet detailing the study aim, expected duration, data storage, and the named investigators. Participants were also informed on the information sheet that any personal data would only be accessible by approved members of the study team and deleted in accordance with the data policy outlined in the information sheet. After reading this, the database hosted the consent form, which had to be fully completed before access to the survey questions was granted via the REDCap system. Completion of consent and the survey was voluntary. At the end of the survey, participants were given the opportunity to be entered into a prize draw to receive either a possible £50 voucher (US$68; 1 was available) or £25 voucher (≈ US$34; 2 were available). They were also asked if they would like to be contacted to participate in a further focus group. Participants could not review their answers or log back in and change them. Only the named study investigators had log-in details to the REDCap survey to protect data privacy. Downloaded data were password-protected and only available to the study team.

#### Analysis

Descriptive statistics were reported for individual survey items. Free text items were analyzed using an inductive approach to identify key themes surrounding participants’ experiences and perceptions of industry-academic partnerships [17].

### Stage 2: Focus Group

#### Recruitment and Participants

Survey participants who indicated interest in participating in further group discussions about the survey findings and the barriers and facilitators to industry-academic partnerships were invited to join a web-based focus group. Those who selected “yes” to this question received a personalized email containing a link to a participant information sheet and consent, providing them with the opportunity to take part. Consent forms had to be completed before the focus group started.

#### Instruments

The focus group topic guide was informed by the survey findings, PPI discussions, and the relevant literature in this field [6,10]. Prompts were developed for the key topic areas, to be used if needed to promote discussion. Prompts were developed based on themes emerging from earlier survey responses and relevant literature, with open-ended questions such as “What would motivate your organization to consider collaborating with academic researchers in the future?” designed to encourage reflection and dialog.

#### Procedure

The focus groups were conducted and recorded using Microsoft Teams, during standard UK working hours. Participants were contacted before the focus group with a short description of each member of the study team who would be attending the focus group as a way of introduction. The focus groups were led by author SSH (PhD), who has experience in conducting remotely delivered focus groups and has completed training courses in qualitative research. SSH is a female researcher who was employed as a Senior Research Fellow at the University of Nottingham at the time of data collection. SSH has 12 years of experience working in academic research and trials. KMP (BA Hons), a female Senior Project Manager employed by the University of Nottingham, assisted with initiating and following up on discussion points where needed. KMP has 17 years of experience working in the industry. OH, a female Research Assistant at the University of Nottingham, supported note-taking throughout the focus group discussions. Focus groups lasted approximately 90 minutes.

#### Analysis

Focus groups were recorded and transcribed using the built-in function in Microsoft Teams. Transcripts were reviewed by OH for accuracy and cross-checked against written notes and the recording of the discussions, where necessary. Transcripts were independently analyzed by 2 researchers (SSH and OH) using an inductive approach, with decisions reviewed, and disagreement resolved through discussion. The researchers conducted open coding to identify recurring concepts and patterns. These initial codes were then grouped into broader categories through discussion, allowing themes to emerge organically from the data rather than being imposed a priori. The researchers met regularly to compare coding decisions, resolve discrepancies, and refine the thematic structure. A third researcher (CLH) oversaw this process and supported discrepancy resolution. This process led to the identification of key themes. A coconstructivist epistemology was operationalized through engagement with participants, allowing for clarification and expansion of ideas, and through collaborative analysis involving multiple researchers. This approach supported the emergence of insights through thematic synthesis rather than predefined coding. For instance, when one participant described inefficiencies in academic meetings, others built on this by sharing contrasting experiences, which collectively shaped the theme “Collaboration Models”.

### Stage 3: Workshop

#### Recruitment and Participants

Nine experts, with varying experience working with mental health industry partners, researchers, clinicians, and PPI members were sent a personalized email invitation to participate in a workshop to review and reach agreement on the key statements to form the Principles of Industry-Academic Partnerships (PIP) guidance. The experts were selected by the study team (authors of this paper) based on the individual’s experience and relevance to the study aims. Selection criteria included: (1) recognized expertise in digital mental health (eg, through innovations, publications, or leadership roles), (2) professional or lived experience in the UK context, and (3) to ensure representation across clinical, academic, public, and industry sectors. Consent was not required to participate in the workshop since there was no formal data collection or analysis procedures and recordings were not taken. This was approved by the ethics committee.

#### Instruments

Before the workshop, the study team drafted the PIP guidance statements based on the findings from the survey and the focus group. The draft statements were circulated to the workshop attendees for review and familiarization 2 weeks before the meeting.

#### Procedure and Analysis

The half-day workshop was conducted using Microsoft Teams software. The workshop was co-facilitated by the study leads (CLH, SSH). Each guidance statement was presented and discussed in turn. The workshop was not recorded with key discussions minuted (OH). No formal analysis procedures were implemented. Discussions in the workshop were centered around making minor refinements to the guidance statements, with no polarizing opinions. Notes from the discussions were used to refine and finalize the PIP guidance statements.

Integration of findings across the 3 stages was achieved through a sequential process. Insights from the survey (Stage 1) were used to inform the focus group topic guide (Stage 2), ensuring that qualitative discussions were built directly on the quantitative findings. Themes emerging from the focus groups were then synthesized and used to draft preliminary guidance statements. These statements were reviewed and refined during the workshop (Stage 3), where participants validated, expanded, or restructured them based on their collective expertise. This triangulated approach ensured that the final PIP guidance was grounded in both empirical data and stakeholder consensus.

### Ethical Considerations

Ethical approval for the study was granted by the University of Nottingham, Faculty of Medicine and Health Sciences Research Ethics Committee, FMHS 298‐0924 approved on October 4, 2024. Informed consent was obtained by a consent form hosted on RedCap before undertaking any study activities. The consent form included a statement on privacy and confidentiality of participant data. Participants in the focus group and workshop were given a £50 (US $68) voucher to compensate for their time.

## Results

### Results of Patient and Public Involvement

PPI input led to a more accessible and relevant survey, with improved clarity and framing of questions. Their contributions shaped the focus group topic guide to better reflect public concerns and lived experience. Through the coproduction workshop, PPI members helped refine and agree upon the final statements, ensuring the guidance was grounded in values relevant to a mental health population.

### Stage 1: Survey Findings

#### Sample Characteristics

The sample characteristics are described in Table 1. A total of 22 people completed the survey. It is not possible to know the exact response rate as study flyers were posted in the conference center at the 2024 Giant Health Conference. However, 62 people were approached to complete the survey directly via email, 26 of whom were contacts known to the study team.

**Table 1.** Demographic and background characteristics of the company (N=22).

Demographic and background items	Count, n (%)	
Type of organization		
Small-medium enterprise (SME)	14 (64)	
Start-up	7 (32)	
Large corporation	1 (5)	
Location		
London	8 (36)	
North West England	3 (14)	
East of England	3 (14)	
North East England	2 (9)	
East Midlands	2 (9)	
Yorkshire and the Humber	1 (5)	
South West England	1 (5)	
Wales	1 (5)	
Other (remote company)^a^	1 (5)	
Role within company		
Founder or cofounder	8 (37)	
Marketing or sales lead	5 (23)	
CEO or executive	4 (18)	
Research and development lead	3 (14)	
Product manager	1 (5)	
Other (chief of staff)	1 (5)	
Years in operation (years)		
More than 10	9 (41)	
7‐10	2 (9)	
4‐6	8 (36)	
1‐3	3 (14)	
Length of time in mental health industry (years)		
More than 10	4 (18)	
7‐10	2 (9)	
4‐6	9 (41)	
1‐3	6 (27)	
Less than 1 year	1 (5)	

a A remote company is a company where employees work in other locations rather than a traditional office space, such as in their own home or coworking spaces.

The majority of the sample was made up of SMEs, accounting for 64% (14/22), with only one company identifying as a single large corporation (1/22, 5%). The companies were primarily based in London (8/22, 36%) but included representation from various regions in England. Founders or cofounders make up the largest role category (8/22, 36%), with marketing and sales leads and CEOs and executives also significantly represented. Most companies had been in operation for over 10 years (9/22, 41%) or between 4 and 6 years (8/22, 36%). In terms of experience in the mental health industry, 41% (9/22) had been involved for 4‐6 years, with smaller proportions having over 10 years or less than 1 year of experience.

Companies primarily offered digital mental health interventions for both adults and children (11/22, 50%). Most products were in the postmarket or scaling stage (12/22, 55%) and funding was predominantly from grants (16/22, 73%), followed by revenue and sales (12/22, 55%) and venture capital (11/22, 50%). The most addressed mental health conditions were anxiety (18/22, 82%) and depression (15/22, 68%). The characteristics of the interventions are summarized in Table 2.

**Table 2.** Characteristics of the main digital mental health intervention (N=22).

Product characteristic	Count, n (%)	
Target age for the product		
Both adults and children and young people	11 (50)	
Adult	8 (36)	
Children and young people	3 (14)	
Stage of product readiness^a^		
Post market or scaling	12 (55)	
Prototype	6 (27)	
Market Launch	6 (27)	
Beta-testing phase	3 (14)	
Primary sources of funding^a^		
Grants (government or private)	16 (73)	
Revenue and sales	12 (55)	
Venture Capital	11 (50)	
Personal or family funds	5 (23)	
Crowdfunding	0 (0)	
Other-internal company R&D funding	1 (5)	
Type of mental health issue product is aimed at^a^		
Anxiety	18 (82)	
Depression	15 (68)	
General well-being	10 (46)	
Stress management	8 (36)	
Developmental disorders	6 (27)	
Sleep disorders	3 (14)	
Substance use	1 (5)	
Other - body image	1 (5)	
Other–pain	1 (5)	
Other–triage	1 (5)	
Other-functional neurological disorder	1 (5)	

a denotes participants could tick more than one box and thus answers will not add up to 100% or N=22.

#### Collaborating With Health Care Professionals, Patients, and Public Members

All our participants reported collaborating with health care professionals at least once, with half (11/22, 50%) stating they had collaborated over 6 times, and the remaining half reported collaborating less than 6 times. The majority of the participants reported feeling completely or very confident in collaborating with health care professionals (17/22, 77%), with the remainder reporting moderate (2/22, 9%) or slight confidence (3/22, 14%).

Only one of the participants reported having never worked with PPI members. The rest of the respondents reported having engaged with PPI as part of their product development (14/22, 64%), evaluation (15/22, 68%), or implementation (10/22, 46%).

Participants were asked to leave free text comments to explain how they had been involved in PPI. Responses included involvement in focus groups or workshops (7/22, 32%), testing the product (2/22, 9%), developing and piloting the product (2/22, 9%), and involvement in trials (2/22, 9%), although they did not clarify the exact tasks or purpose of this involvement. Participants were also asked to describe the benefits of PPI; answers included to validate the product and innovation (8/22, 36%), support real-world understanding of the product (6/22, 27%), to aid product development (5/22, 23%), and to improve the product or service (2/22, 9%).

#### Collaborations With Academics

Only one participant had never collaborated with academics previously. This participant was directed to a different set of questions via survey logic (see Appendix C for survey items in Multimedia Appendix 1). This one participant was asked further questions about why they had not engaged with researchers. They noted that the key barrier was a lack of clear benefits, concerns of intellectual property, and their eagerness to publish results. They also reported that the high costs associated with trials were a barrier to engagement, as well as the complex and time-consuming nature of academic collaboration.

Out of the remaining 21 participants, 10/21 (47%) had collaborated with academics one to two times, 6 (29%) had collaborated between 3 and 5 times, and 5/21 (23%) had collaborated 6 or more times.

The majority 14/21 (67%) reported seeking support from external organizations to initiate a collaboration with academics. Sources of this support included the National Institute for Health Research MindTech, a national center focusing on the development, adoption, and evaluation of new technologies, and Health Innovation Networks, a UK-based government-funded infrastructure that brings together the NHS, industry, academic, third sector, and local organizations to support early adoption of innovations into practice. Other sources of support included Innovation UK; Wellcome Trust, who are UK-based research funders; and more broadly, responses included “NHS” and “Universities.”

Most respondents reported positive experiences of collaborating with academics, with 19/21 (90%) agreeing that their experience was positive and 18/21 (86%) feeling communication was clear and effective. Similarly, 17/21 (81%) found the process valuable for developing the intervention. Expectations alignment was slightly lower, with 14/21 (67%) agreeing that expectations aligned. Just over half of the participants (11/21, 52%) felt that the pace at which academic evaluation is conducted hindered their business. Despite this, the same number of participants, 11/21 (52%), felt that cultural differences between academia and industry were not a barrier to collaboration, and the sample was divided as to whether regulatory and ethical procedures had caused challenges for their product’s evaluation.

The majority of participants (18/21, 86%) reported being satisfied with the outcome of their academic collaborations, while 3/21 (14%) felt neutral about their experiences. However, all 21 respondents said that they would be willing to engage in future collaborations with academic researchers again.

Participants were asked 3 open-text questions to understand challenges and advantages to collaborating with industry. The primary purpose of these questions was to inform the topic guide for the focus groups. However, for completeness, we summarize the key findings from these.

With reference to challenges, the key barriers were centered around costs (such as overheads and on-costs) and timeframes (20/21, 95%). Participants noted that university procedures and bureaucracy often meant delayed timelines, with one participant noting that this is not the same with private companies.

*Private companies tend to be more flexible and wish some modifications to planned projects based on preliminary outcomes and business needs while academic researchers wish to stay on initial plans understandably.*

[Participant ID 71]

Noted key advantages of collaboration included support on study designs and ethics (7/21, 33%), and evidence that the product works, including increased reputation and credibility (10/21, 48%).

When asked what support would be needed to support academic collaborations, responses included more funding (8/21, 38%), support in finding academic experts (6/21, 29%), universities having a better understanding of how to work with industry (4/21, 19%), and better PPI support (3/21, 14%).

#### Randomized Controlled Trials

Participants were also asked if they had conducted a randomized controlled trial for their product partnering with an academic. Three respondents had not engaged in a randomized controlled trial. Out of the remaining 18, all (18/18, 100%) reported strongly agreeing or agreeing that their organization was adequately involved in the design of the trial and trial process. The majority (17/18, 94%) reported that the outcome measures used in the trial were relevant to the product’s real-world application, with one responding “neutral.”

### Stage 2: Focus Groups Findings

A total of 5 participants took part in the focus groups. Participants in both the first focus group (n=3) and the second focus group (n=2) had experience of engaging in industry-academic research partnerships.

Four major themes were evident in the first focus group. The second focus group provided further enrichment to this, but no additional themes were identified. The themes formed the basis of study team discussions to develop recommendations to support industry-academic partnerships, which were evaluated in the workshop stage of the project.

The 4 major themes evidenced in the focus groups were: Advantages, Industry versus academic culture, Collaboration models, and Structural issues within the universities. These themes and their subthemes are reported below.

#### Advantages

This theme was comprised of 3 subthemes: quality and credibility; knowledge transfer and talent development; and patient and public involvement, access, and management.

Academic work was perceived as offering a different kind of quality and credibility with more theoretical rigor and scrutiny, as well as offering an independent form of evaluation.

*It’s a different kind of quality, right? It comes from a place of theory and a place of scrutiny, as opposed to industry, which is a different kind of high quality*

[P01]

Industry partners value the “academic stamp of approval” for enhancing the credibility of their products.

*It may be that we need we kind of need their logo or we need the university, especially in the US it’s like we need an academic stamp of approval from a certain university and that’s how it works*

[P03]

Partnerships with universities provide opportunities for knowledge transfer and talent development.

*So by design there’s an element of upskilling of other people within the team to learn more about these kind of, you know, academic methods for example*

[P02]

Industry-academic partnerships support talent development, allowing researchers to experience industry environments and vice versa. This can benefit individual career development as well as positively contribute to the wider partnership in terms of providing a go-to person who can act as a translator and/or mediator between the 2 partners. Identification of this individual was also noted to improve the longevity of industry-academic partnerships. Participants discussed the benefits of Knowledge Transfer Partnerships (KTPs), a scheme in which a graduate works at both a company and university to share knowledge and facilitate company development.

*Just in terms of like different speaking different languages, I think that’s something that we've really valued, KTPs for is that in some way the person working on the KTP is an interpreter between academia and like industry*.

[P02]

Partnering with academics was deemed valuable in terms of providing access to and support from PPI groups. Participants recognized that PPI was important to product development, but that the industry often lacked the resources in terms of connections, as well as the face value trust which is needed to engage patients and the public.

*I definitely think this is where the university plays into its strengths because ultimately, you know, a patient is much more likely to engage with stakeholder meetings with a university than they might be a small business just because. There’s an element of trust there or an element of reputation there*.

[P01]

#### Industry vs Academic Culture

This theme was comprised of 3 subthemes: incentives and drivers, differences in timescales and decision making, and rigid versus dynamic mindsets.

Participants commented that the different incentives and drivers for industry and academic partners can create tension in the relationship, particularly in terms of the fact that industry partners may need to “fail fast” to cut their losses, whereas academic partners are in for the long haul to the pursuit of knowledge acquisition.

*In our kind of setting, you know, you might want to fail fast if something isn't going to work out. You want to find that out quickly and stop working on it and move on. And that’s maybe a bit of tension within an academic partnership where it might be something that you have set up that we're going to run for a number of years*

[P02]

*It takes a fair degree of patience on both sides and there’s this inherent tension. I think we've noticed between the different incentives that each party is operating under in that stands to risk jeopardising the relationship, or it certainly puts tension on the relationship at various moments throughout the life of that partnership*.

[P05]

Cultural differences were also discussed frequently in terms of differences in timescales and decision making. Participants commented that academics operated over months and years as opposed to days and weeks, and that setting deadlines was important for achieving a positive working relationship.

*We're working companies who are reactive all the time. So we're constantly getting answers from our team about what’s happening and we're working in an immediate culture. So it’s the contrast then where you might e-mail someone and it might be, you know seven days before you get a reply and it so it’s so it’s like a feels quite jarring*.

[P04]

Academics were viewed as being rigid in their approach to an evaluation, with a clear start and end point. In contrast, industry is often more dynamic and used to adapting to situations as they arise, even if that means making substantial changes to the project plan (protocol).

*Do they (industry partners) understand that it will be a bit more rigid usually like once we start it’s quite like we're going down the same kind of road, there are some kind of like pivot points that if you've missed those you've pretty much kind of you're going to continue down that road……I think there’s needs to be a bit of balancing of like actually what would work so they (academics) need to be quite pragmatic and balancing their expectations with that can be a bit challenging*.

[P03]

#### Collaboration Models

This theme was comprised of 3 subthemes, efficiency and key people, initial challenges and establishing relationships, and the role of facilitators. The theme focuses on ways of working between academia and industry.

Participants reflected on the number of academic staff that are involved in meetings and collaboration in relation to efficiency and key people*,* with current academic practice considered an often inefficient and ineffective model of collaboration. Whereas academics often encompass a more inclusive model in their way of working, industry feels only key people are required, and that involving too many people can slow down progress.

*Industry has become very good at only having the people that need to be in the room in the room. And I think that’s one of the key things is the more hands you have on this, the slower things that just get generally going*

[P01]

Interestingly, participants felt that more senior academics were often not required. This is in contrast to a traditional academic collaboration, whereby senior academics, such as Professors, have a higher status and are typically considered critical in key meetings. Conversely, the industry placed more emphasis on having attendees who would be able to action tasks, which they noted might be more junior staff.

*If you go kind of too senior, it means that they just don't have enough time, they're not really responding. You're chasing things from them and that’s when it breaks down.*

[P03]

Participants reported that establishing relationships with academics was often the hardest part of collaboration. Some noted that initial relationships are often formed by industry reaching out to individuals, or via networking and conferencing events.

*I think actually getting to a point where you can work with researchers is often the hard part when working with academia, the researchers are typically very easy to work with once we've kind of established the working arrangement.*

[P01]

The importance of selecting the right partnership was mentioned, with emphasis placed on having visions and goals that aligned with each other. Industry representatives acknowledged that there are likely to be times in research where honest communication is required, and it’s important to have academic partners with whom you are able to have open conversations.

*Is there a good like personality fit because you're going to need to work with them and you're going to have difficult conversations.*

[P03]

The importance of the role of facilitators in brokering that early relationship and continuing to support throughout the collaboration was also noted. Most respondents noted the importance of networks such as the Health Innovation Network or KTPs. KTPs, although different in remit and scope to the HIN, also connect businesses with academic experts to support innovation.

*The HIN has been fundamental…they’re a nice broker in that relationship*

[P01]

Participants noted that these facilitators are particularly useful in interpreting academic language.

*In terms of speaking different languages, that’s something we’ve really valued KTPs for…the KTP is an interpreter between academic and industry*

[P02]

The importance of also having proacademic employees within the industry business was also noted, with participants also noting having an academic champion from within can be useful, particularly if other members have had negative experiences of partnering with academia.

*Definitely had situations in the past with people who are quite negative about academic relationships… so having someone in the in the company, that’s a good bridge would be useful.*

[P03]

#### Structural Issues Within the Universities

This prominent theme highlighted core structural issues with the university that resulted in significant barriers to collaboration, including cost barriers and bureaucratic inefficiencies.

Participants noted cost barriers*,* with SMEs often operating on small budgets without the capital to invest significantly in research. They noted that the costs of academia often precluded the ability to collaborate and that more competitive pricing was often offered by consultants. Although consultants were considered to still offer many of the advantages associated with academic institutions, including good networks and knowledge of study design and publication.

*And our experiences that has just blown out the water in terms of what we can afford. So we have to go to someone else, a consultant that’s working privately*

[P04]

Even when industry and academics partnered to fund the work through external grants, there was still a feeling of resentment toward the costs associated with academic partnership, most notably, the staff overheads.

*the overhead spend on academic resources is outrageous. We're working on another project and half of it [THE COSTS] went on university overheads. I just think that is unacceptable*

[P01]

University bureaucratic inefficiencies contributed to inefficient processes and decision-making, which led to delays in initiating collaborations and applying for grants.

*from a company perspective, we would turn a grant around a grant application around in two to three weeks…the universities we've tried to collaborate with can't work in that sort of time scale.*

[P01]

It was noted that this was at least in part driven by the large-scale nature of university organizations, alongside the nature of universities to be “risk averse” and requiring the approval and sign-off from multiple people, slowing decision-making and project initiation.

Challenges with contracting and IP were prominent issues in the focus groups, which were partially driven by lengthy and complex internal governance structures. The contracting discussions can take months due to back-and-forth negotiations over templates, agreements, and responsibilities.

*academia is often very slow in contracting. The governance is complicated*

[P05]

Issues with IP were considered to contribute to this, and it was noted that, particularly, smaller universities, or those not used to industry collaborations, may not be as adept at navigating IP.

*I think ultimately universities get tied up in IP…. whereas actually it’s not often that complicated*

[P01]

Participants also noted that there is often confusion and tension when determining which templates to use for drafting these agreements on IP and contracts. This lack of clarity can create delays and frustration, especially when different teams have different expectations about the terms.

*there’s like a little bit of a back and forth between whose agreements do we use? Whose templates do we use for the IP? Are we all agreeing on that and it’s and the actual our team and the researchers aren't that bothered. It’s just the two entities that are kind of battling back and forth sometimes*

[P03]

### Stage 3: Workshop Findings

The workshop members reviewed and provided feedback on 15 guidance “action” statements, which were developed by the study team based on the survey and focus group findings. The action statements were initially separated under the themes derived from the focus groups.

Discussions in the workshop included whether implementing this guidance would result in meaningful change and the feasibility of implementation. As a result of these discussions, the 15 action statements were modified. These modifications included separating the statements into broader headings, which were more reflective of the research roadmap (project initiation, defining the scope and agreements, project execution, and promoting sustainability). As well as making minor alterations to the wording of the action statements, some were merged together and/or segmented into separate actions. This resulted in a final PIP guidance document, comprised of 14 key actions (See Appendix D in Multimedia Appendix 1). The PIP guidance is presented in a text and infographic version to provide both detailed and rapid (eg, an aide-mémoire) support to teams depending upon the situation or context.

The PIP guidance document provides a structured framework to supporting effective industry-academic partnerships in mental health research. Key actions are organized into 4 phases: project initiation, defining the scope and agreements, project execution, and promoting sustainability. The guidance emphasizes early engagement, cost-effective funding models, streamlined contracting, and clear intellectual property agreements to facilitate smoother partnership setups. It also highlights the importance of structured project management, effective communication strategies, and fostering long-term sustainability through knowledge exchange and talent development. By addressing common challenges such as balancing speed with rigor or navigating cultural differences, the PIP guidance aims to improve collaboration and maximize research impact.

## Discussion

### Overview

We used the digital mental health field as a case example to explore how industry-academic partnerships can be improved, from the perspective of industry partners. We conducted a web-based survey and focus groups to examine digital mental health companies’ experiences and perceptions of collaborating with academics to evaluate their products. Insights from these activities informed the development of practical guidance to support more effective partnerships. Our findings revealed that overall, the industry valued academic partnerships, particularly for the credibility, rigor, scientific knowledge, and access to PPI that they provide. However, significant barriers to engagement were noted, particularly around the financial costs of collaboration with academics and the differences in timescales for delivery. Through discussions with our experts (including people with lived experience of mental health conditions and those working in mental health through a clinical and industry-focused role), these challenges were positioned into principles for supporting positive industry-academic collaborations. These principles have been developed based on experiences of digital mental health industry partners (see Textbox 1) but are likely to apply more broadly outside of the field of mental health.

Textbox 1. **Case study illustrations.**

Case Example 1. One small to medium enterprise (SME) in our sample developed a digital CBT (Cognitive Behavioral Therapy) app for adolescents with anxiety. They partnered with an academic team to conduct a feasibility trial. The collaboration benefited from academic expertise in safeguarding and ethics, and access to a university-affiliated patient and public involvement (PPI) group that helped adapt the app’s language and content. However, delays in contracting and differing expectations around trial timelines created tension. Applying the Principles of Industry-Academic Partnerships (PIP) guidance, particularly around early alignment of expectations and streamlined governance, could have mitigated these challenges.

Case Example 2. A company that was developing a digital intervention for stress management partnered with an academic team to evaluate the intervention’s feasibility and acceptability. While the collaboration was productive overall, the company noted inefficiencies in academic meeting structures, where large numbers of attendees slowed decision-making and diluted accountability. In contrast, the company’s internal culture prioritized lean teams and rapid iteration. This mismatch led to frustration and delays in progressing the evaluation. The PIP guidance’s emphasis on defining collaboration models early—particularly around team roles, decision-making authority, and meeting structures—could have helped align expectations and improve operational efficiency. This example illustrates how differences in working culture can impact progress, and how structured guidance can support smoother, more agile partnerships in mental health research.

### Principal Findings

The focus group discussions identified 4 major themes—Advantages, Industry versus academic culture, Collaboration models, and Structural issues within the universities—each highlighting key factors influencing industry-academic partnerships.

Industry partners recognized the value of academic collaborations in enhancing credibility, facilitating knowledge transfer, and supporting talent development. However, cultural differences, particularly in incentives, decision-making timelines, and flexibility, often created tensions. Industry operates with a “fail fast” mindset, whereas academia prioritizes long-term knowledge generation, leading to challenges in aligning expectations. Efficient collaboration models were seen as crucial, with industry favoring smaller teams and actionable engagement, while academia often involved broader participation. Establishing partnerships was noted as a key hurdle, emphasizing the role of facilitators such as KTPs in bridging communication gaps. Structural barriers within universities, including high costs, bureaucratic inefficiencies, and intellectual property disputes, further complicated partnerships. These findings underscore the need for universities to streamline administrative processes, adopt more flexible collaboration models, and enhance support mechanisms to foster stronger, more efficient industry-academic relationships. Addressing these challenges could lead to more impactful partnerships that better translate academic research into real-world applications. In addition, issues with IP indicate the importance of having a department within a research institution, such as a Technology Transfer Office. These departments manage the IP generated by researchers and support the commercialization of research findings. Such roles are critical to ensure fair agreements on revenue sharing, IP rights, and protecting the assets of partners.

Survey respondents commonly reported involving PPI in their research and design, primarily through focus groups or meetings. However, fewer described engagement in product testing, development, or trials, suggesting a limited understanding of what constitutes meaningful PPI. A one-off focus group, for instance, does not equate to fully integrating PPI contributors as active members of the research and development team, an approach more consistent with coproduction. This highlights the need for clearer guidance and support for industry partners on effective PPI implementation and best practices. These findings are especially pertinent to sectors where trust, safeguarding, and stigma are critical considerations, such as in mental health. In this field, meaningful PPI is not just a methodological enhancement but a necessity for ensuring interventions are acceptable, ethical, and responsive to the needs of vulnerable populations. Industry partners in our study valued academic collaboration for access to established PPI networks, which can play a vital role in shaping mental health research that is both inclusive and impactful.

### Comparison With Prior Work

These findings support a recent systematic review in this area which highlighted issues with communication (collaboration models) and identified some cultural differences, particularly in terms of goals of the partnership [6]with industry-academic partnerships in general (not specific to the mental health field). However, unlike the previous research [6], the PIP guidance is the first co-developed guidance, shaped by the lived experiences of industry partners. Although small scale, by combining quantitative (survey) and qualitative (focus groups and workshops) research methods and talking directly with industry partners, we can better understand how to support positive industry-academic collaborations. Developed within the context of digital mental health, the guidance addresses cross-cutting structural and relational factors that are broadly applicable across sectors. Its emphasis on actionable strategies and stakeholder co-creation distinguishes it as a practical tool for enhancing the impact and sustainability of cross-sector partnerships.

### Strengths and Limitations

Given the early stage of inquiry in this area, our findings provide valuable insights into industry-academic collaborations which will not only directly inform practice, through implementation of our proposed PIP guidance, but also inform future research efforts in this area. Our study is strengthened by our coproduced approach to survey and focus group item generation. We sought opinions from industry engagement experts, as well as patient and public involvement members who had lived experiences of mental ill health and supporting industry partners in evaluating their products. Our mixed-methods approach, combining quantitative responses from a broader survey sample and in-depth qualitative responses, enabled us to explore experiences in more detail, which is important given the early stage of inquiry in this field.

Nonetheless, our findings should be interpreted in line with the limitations of our relatively small sample, which prevents us from making subgroup comparisons, and how this sample was recruited. Although we advertised the study via social media, including via the Health Innovation Network, the Institute of Mental Health, and the work accounts of our team, we did not receive any uptake from these methods. The lack of uptake via social media may demonstrate the difficulties that academics might face when engaging with the digital mental health industry and highlight the importance of having a personal connection and being flexible to trying novel methods of recruitment. Our sample was primarily comprised of individuals who were emailed by members of the National Institute of Health Research (NIHR) MindTech Health Research Centre (HRC) team, the study authors, and the Center for Healthcare Equipment and Technology Adoption (CHEATA). This may also reflect that most of the sample had previously collaborated with academics, which is unlikely to accurately reflect the real percentage of digital mental health companies that have partnered with academics. However, we were also successful in recruiting participants via snowballing (encouraging participants to forward survey links to their peers and competitors) and also by networking at a national conference “Giant Health” in December 2025, a conference aimed at industry and NHS health care innovation in the United Kingdom.

To mitigate the potential responder bias, focus groups were held by members of the study team that had not previously worked with the industry partners. Participants were also informed that their personal or identifiable information would be removed before wider sharing within the team; however, we cannot rule out that this may have influenced responding. That aside, the focus groups did reveal significant barriers to collaboration, which may indicate that participants were responding candidly.

Most of our sample identified as an SME, which potentially reflects the status of most of the digital mental health industry; however, our findings may not be generalizable to other companies, particularly large corporations, which were poorly represented in our sample. In addition, while our sample included companies working across a diverse range of mental health areas, the guidance developed in this study is intended to address overarching structural and relational factors in industry, academic partnerships. Future research may be needed to adapt and tailor these principles to specific mental health contexts or diagnostic categories. Furthermore, our participants were drawn from the United Kingdom, and further research across different countries is required to understand the cultural context of these findings. Despite these limitations, our study provides a novel insight into a currently overlooked area. We hope the findings presented here and our guidance document will be useful to generate further research, including exploring the perceptions of academics, larger corporations, and those who have not engaged with academics, and facilitate research with larger sample sizes. Given the guidance was developed from the viewpoint of the industry partner, we believe at this stage in its development academic partners are likely to gain the greatest benefit from it. Future investment in this area should focus on academic needs and experiences. The guidance document could be used within Tech Transfer and Research and Knowledge Exchange (RKE) groups to support research groups, across career stages, as they embark upon research collaborations with industry partners.

### Patient and Public Involvement Reflection

PPI had a meaningful influence on the study, contributing to both the design and interpretation of key components of the guidance. Our PPI members had direct experience with industry collaborations, which facilitated meaningful and engaged discussions. However, broader representation might have enriched the guidance further. Navigating discussions around industry involvement required careful facilitation to ensure contributors felt comfortable expressing concerns. Future research should consider how to support and diversify PPI contributors to ensure inclusive and informed dialog across stakeholder boundaries.

### Conclusion

While industry partners identified several advantages of working with academics to develop and evaluate digital mental health interventions, they also reported significant barriers to collaboration. Many of these barriers centered around university processes, such as contracting and costings. It is important that universities address these structural and cultural barriers in order to create more effective and sustainable collaborations. The PIP guidance developed in this study offers a practical framework to support collaborations and improve mental health research capacity by streamlining institutional processes, aligning expectations, and improving collaboration models. While the guidance was developed in the context of digital mental health, it addresses cross-cutting issues that are relevant across sectors. As such, it provides a generalizable foundation for strengthening industry-academic collaboration and driving innovation in fields where interdisciplinary partnerships are key to addressing complex societal challenges.

## Supplementary material

10.2196/77439 Multimedia Appendix 1 Principles of Industry-Academic Partnerships guidance development.

## Abbreviations

CEO: Chief Executive Officer
CHEATA: Center for Healthcare Equipment and Technology Adoption
CHERRIES: Checklist for Reporting Results of Internet E-Surveys
GRIPP2-SF: Guidance for Reporting Involvement of Patients and the Public-Short Form
HIN: Health Innovation Network
IP: intellectual property
KTP: Knowledge Transfer Partnerships
NHS: National Health Service
PIP: Principles of Industry-Academic Partnerships
PPI: Patient and Public Involvement
REDCap: Research Electronic Data Capture
SME: small-medium enterprise
