# Supplementary material for: Principles of Industry-Academic Partnerships Informed by Digital Mental Health Collaboration: Mixed Methods Study
Source: JMIR Ment Health. 2025 Sep 10;12:e77439. doi: 10.2196/77439 (PMC12422718; doi:10.2196/77439)
Supplement: Multimedia Appendix 1 [file mental-v12-e77439-s001.docx]

**PIP Guidance Development – Appendices**

**Appendix A - Guidance for Reporting Involvement of Patients and the Public- Short Form (GRIPP2)**

| **Section and topic** | **Item** | **Reported on page No** |
| --- | --- | --- |
| 1: Aim | Report the aim of PPI in the study | P5 |
| 2: Methods | Provide a clear description of the methods used for PPI in the study | P5 |
| 3: Study results | Outcomes—Report the results of PPI in the study, including both positive and negative outcomes | P9 |
| 4: Discussion and conclusions | Outcomes—Comment on the extent to which PPI influenced the study overall. Describe positive and negative effects | P24 |
| 5: Reflections/critical perspective | Comment critically on the study, reflecting on the things that went well and those that did not, so others can learn from this experience | P24 |

**Appendix B - Checklist for Reporting Results of Internet E-Surveys (CHERRIES)**

| ***Checklist Item*** | ***Explanation*** | ***Page Number*** |
| --- | --- | --- |
| Describe survey design | Describe target population, sample frame. Is the sample a convenience sample? (In “open” surveys this is most likely.) | Page 5 |
| IRB approval | Mention whether the study has been approved by an IRB. | Page 4 |
| Informed consent | Describe the informed consent process. Where were the participants told the length of time of the survey, which data were stored and where and for how long, who the investigator was, and the purpose of the study? | Page 6 |
| Data protection | If any personal information was collected or stored, describe what mechanisms were used to protect unauthorized access. | Page 6 |
| Development and testing | State how the survey was developed, including whether the usability and technical functionality of the electronic questionnaire had been tested before fielding the questionnaire. | Page 5-6 |
| Open survey versus closed survey | An “open survey” is a survey open for each visitor of a site, while a closed survey is only open to a sample which the investigator knows (password-protected survey). | Page 6 |
| Contact mode | Indicate whether or not the initial contact with the potential participants was made on the Internet. (Investigators may also send out questionnaires by mail and allow for Web-based data entry.) | Page 6 |
| Advertising the survey | How/where was the survey announced or advertised? Some examples are offline media (newspapers), or online (mailing lists – If yes, which ones?) or banner ads (Where were these banner ads posted and what did they look like?). It is important to know the wording of the announcement as it will heavily influence who chooses to participate. Ideally the survey announcement should be published as an appendix. | Page 5 |
| Web/E-mail | State the type of e-survey (eg, one posted on a Web site, or one sent out through e-mail). If it is an e-mail survey, were the responses entered manually into a database, or was there an automatic method for capturing responses? | Page 5 |
| Context | Describe the Web site (for mailing list/newsgroup) in which the survey was posted. What is the Web site about, who is visiting it, what are visitors normally looking for? Discuss to what degree the content of the Web site could pre-select the sample or influence the results. For example, a survey about vaccination on a anti-immunization Web site will have different results from a Web survey conducted on a government Web site | Page 5 |
| Mandatory/voluntary | Was it a mandatory survey to be filled in by every visitor who wanted to enter the Web site, or was it a voluntary survey? | Page 5-6 |
| Incentives | Were any incentives offered (eg, monetary, prizes, or non-monetary incentives such as an offer to provide the survey results)? | Page 6 |
| Time/Date | In what timeframe were the data collected? | Page 6 |
| Randomization of items or questionnaires | To prevent biases items can be randomized or alternated. | Not applicable |
| Adaptive questioning | Use adaptive questioning (certain items, or only conditionally displayed based on responses to other items) to reduce number and complexity of the questions. | Page 6 |
| Number of Items | What was the number of questionnaire items per page? The number of items is an important factor for the completion rate. | Page 6 |
| Number of screens (pages) | Over how many pages was the questionnaire distributed? The number of items is an important factor for the completion rate. | Page 6 |
| Completeness check | It is technically possible to do consistency or completeness checks before the questionnaire is submitted. Was this done, and if “yes”, how (usually JAVAScript)? An alternative is to check for completeness after the questionnaire has been submitted (and highlight mandatory items). If this has been done, it should be reported. All items should provide a non-response option such as “not applicable” or “rather not say”, and selection of one response option should be enforced. | Page 6 |
| Review step | State whether respondents were able to review and change their answers (eg, through a Back button or a Review step which displays a summary of the responses and asks the respondents if they are correct). | Page 6 |
| Unique site visitor | If you provide view rates or participation rates, you need to define how you determined a unique visitor. There are different techniques available, based on IP addresses or cookies or both. | NA |
| View rate (Ratio of unique survey visitors/unique site visitors) | Requires counting unique visitors to the first page of the survey, divided by the number of unique site visitors (not page views!). It is not unusual to have view rates of less than 0.1 % if the survey is voluntary. | NA |
| Participation rate (Ratio of unique visitors who agreed to participate/unique first survey page visitors) | Count the unique number of people who filled in the first survey page (or agreed to participate, for example by checking a checkbox), divided by visitors who visit the first page of the survey (or the informed consents page, if present). This can also be called “recruitment” rate. | NA – we didn’t record this |
| Completion rate (Ratio of users who finished the survey/users who agreed to participate) | The number of people submitting the last questionnaire page, divided by the number of people who agreed to participate (or submitted the first survey page). This is only relevant if there is a separate “informed consent” page or if the survey goes over several pages. This is a measure for attrition. Note that “completion” can involve leaving questionnaire items blank. This is not a measure for how completely questionnaires were filled in. (If you need a measure for this, use the word “completeness rate”.) | Page 9 |
| Cookies used | Indicate whether cookies were used to assign a unique user identifier to each client computer. If so, mention the page on which the cookie was set and read, and how long the cookie was valid. Were duplicate entries avoided by preventing users access to the survey twice; or were duplicate database entries having the same user ID eliminated before analysis? In the latter case, which entries were kept for analysis (eg, the first entry or the most recent)? | Page 5-6 |
| IP check | Indicate whether the IP address of the client computer was used to identify potential duplicate entries from the same user. If so, mention the period of time for which no two entries from the same IP address were allowed (eg, 24 hours). Were duplicate entries avoided by preventing users with the same IP address access to the survey twice; or were duplicate database entries having the same IP address within a given period of time eliminated before analysis? If the latter, which entries were kept for analysis (eg, the first entry or the most recent)? | Page 6 |
| Log file analysis | Indicate whether other techniques to analyze the log file for identification of multiple entries were used. If so, please describe. | NA |
| Registration | In “closed” (non-open) surveys, users need to login first and it is easier to prevent duplicate entries from the same user. Describe how this was done. For example, was the survey never displayed a second time once the user had filled it in, or was the username stored together with the survey results and later eliminated? If the latter, which entries were kept for analysis (eg, the first entry or the most recent)? | NA |
| Handling of incomplete questionnaires | Were only completed questionnaires analyzed? Were questionnaires which terminated early (where, for example, users did not go through all questionnaire pages) also analyzed? | Page 5 (NA -everyone completed) |
| Questionnaires submitted with an atypical timestamp | Some investigators may measure the time people needed to fill in a questionnaire and exclude questionnaires that were submitted too soon. Specify the timeframe that was used as a cut-off point, and describe how this point was determined. | NA |
| Statistical correction | Indicate whether any methods such as weighting of items or propensity scores have been used to adjust for the non-representative sample; if so, please describe the methods. | NA |

**Appendix C**

**UNITE Survey Questions**

The following survey questions were administered to participants online via REDCap.

**Demographic Questions**

1. **Organisation Size:** "How many employees work in your organisation?"
   1. 1-10
   2. 11-50
   3. 51-200
   4. 201-500
   5. 501-1000
   6. 1001+
2. **Type of Organisation:** "What type of organisation do you represent?"
   1. Start-up
   2. Small or Medium Enterprise (SME)
   3. Large Corporation
   4. Non-Profit Organisation
   5. Academic Spin-off
   6. Other (please specify)
3. **Years in Operation:** "How many years has your organisation been in operation?"
   1. Less than 1 year
   2. 1-3 years
   3. 4-6 years
   4. 7-10 years
   5. More than 10 years
4. **Geographic Location:** "Where is your organisation headquartered?"
   1. (List of regions in England)
5. **Role in Organisation:** "What is your role within your organisation?"
   1. Founder/Co-Founder
   2. CEO/Executive
   3. Product Manager
   4. Research & Development Lead
   5. Clinical Lead
   6. Regulatory/Compliance Officer
   7. Marketing/Sales Lead
   8. Other (please specify)
6. **Experience with Digital Mental Health:** "How long have you been working in the digital mental health industry?"
   1. Less than 1 year
   2. 1-3 years
   3. 4-6 years
   4. 7-10 years
   5. More than 10 years
7. **Focus of Digital Mental Health Intervention:** "Which area(s) does your digital mental health intervention primarily focus on? (Select all that apply)"
   1. Anxiety
   2. Depression
   3. Stress Management
   4. Substance Use
   5. Sleep Disorders
   6. General Well-being
   7. Other (please specify)
8. **Target Age Group:** "Which age group does your digital mental health intervention primarily focus on?"
   1. Children and young people
   2. Adults
   3. Both children and young people and adults
9. **Stage of Product Development:** "At what stage is your digital mental health intervention currently?" (Select all that apply)
   1. Concept/Prototype
   2. Beta/Testing Phase
   3. Market Launch
   4. Post-Market/Scaling
   5. Other (please specify)
10. **Funding Sources:** "What are your primary sources of funding for your digital mental health intervention? (Select all that apply)"
    1. Venture Capital
    2. Grants (government or private)
    3. Crowdfunding
    4. Revenue/Sales
    5. Personal/Family Funds
    6. Other (please specify)

**Collaboration Questions**

1. **Healthcare Professionals:** "How many times has your organisation collaborated with healthcare professionals (e.g., clinicians) to evaluate your product?"
   1. 0
   2. 1-2 times
   3. 3-5 times
   4. 6+ times
2. **Confidence in Collaboration:** "How confident do you feel in developing collaborations with healthcare professionals?"
   1. Not at all confident
   2. Slightly confident
   3. Moderately confident
   4. Very confident
   5. Completely confident
3. **Patient and Public Involvement:** "At what stage(s) in the development of your innovation have you worked with patients and the public?"
   1. At the start (development)
   2. Middle point (evaluating)
   3. End point (implementation)
   4. Have never worked with PPI
4. **Barriers to Patient and Public Involvement:** "If you haven’t involved patients and the public, what prevented you from doing so?"
   1. Lack of opportunity to build relationships with patients and the public
   2. Lack of engagement/communication skills
   3. Not sure about the benefits of working with patients and the public
   4. Other (please specify)
5. **Methods of Patient and Public Involvement:** "If you did work with patients and the public, in what way did you do so? (e.g., reading the study documents, reviewing the study measures)"
6. **Perceived Benefits of Patient and Public Involvement:** "What do you think the benefits of working with patients and the public were? (Open-ended)"
7. **Previous Collaborations with Academics:** "How many times has your organisation collaborated with academic researchers to evaluate your product?"
   1. 0
   2. 1-2 times
   3. 3-5 times
   4. 6+ times

**Core Questions on Experiences Collaborating with Academics**

*Note: These questions are skipped for those who answer ‘0’ to the question above*

**Initiating collaboration:**

1. **“**Did you seek external support to initiate a collaboration with academics/clinical trialists (e.g., MindTech, Health Innovation Network/Academic Health Science Network)”

- Yes (if yes please state who and how you found them)
- No (if not please briefly describe how you started your collaboration)
- Not sure

**Please rate your level of agreement with the following statements:**

Strongly Disagree | Disagree | Neutral | Agree | Strongly Agree

**Experience with Collaboration:**

1. "My organisation has had positive experiences collaborating with academic researchers."
2. "Academic collaborations have provided valuable insights for the development of our digital mental health interventions."

**Communication and Expectations:**

1. "Communication between my organisation and academic researchers has been clear and effective."
2. "Expectations from academic researchers regarding our collaboration were well-aligned with our organisation’s goals."

**Trial and Evaluation Design:**

1. "My organisation was adequately involved in the design of the evaluation or trial process."
2. "The outcome measures used in the trials were relevant to our product’s real-world application."

**Challenges and Barriers:**

1. "Regulatory and ethical considerations posed significant challenges in our collaboration with academic researchers."
2. "Cultural differences between academia and industry have been a barrier to effective collaboration."
3. "The pace of academic research has hindered our ability to meet our business timelines."

**Overall Satisfaction:**

1. "Overall, I am satisfied with the outcomes of our collaborations with academic researchers."
2. "I would be willing to engage in future collaborations with academic researchers."

**Open-Ended Questions**

**Challenges and Advantages Encountered:**

1. "What were the biggest challenges your organisation faced when collaborating with academic researchers? How did these challenges impact your projects?"
2. "What were the biggest advantages your organisation experienced when collaborating with academic researchers? How did these advantages impact your projects?"

**Suggestions for Improvement:**

1. "What support or resources would help your organisation consider academic collaborations for evaluating or trialling your digital mental health intervention?"

**Core Questions for those who have not collaborated with academics**

*Note: these questions will only be answered by those who answered ‘0’ for question 17*

**Prior Collaboration Attempts**

1. "Has your organisation ever considered collaborating with academic researchers to evaluate or trial your digital mental health intervention?"

- No we haven’t ever considered it
- Yes - we explored the idea but did not pursue it

**Perceived Barriers and Challenges**

**Awareness of Opportunities:**

1. "Are you aware of the potential benefits of collaborating with academic researchers to evaluate or trial your digital mental health intervention?"

- Not aware at all
- Somewhat aware
- Fully aware

1. "Would you know who to contact in terms of an academic researchers to evaluate or trial your digital mental health intervention?"

- No
- Yes (if yes please name who you would contact)

**Resource Constraints:**

1. "To what extent do the following factors influence your decision not to collaborate with academic researchers? (Rate each on a scale of 1-5, where 1 = Not at all and 5 = To a great extent)"

- Lack of funding/resources
- Time constraints
- Limited personnel to manage the collaboration
- High costs associated with academic trials

**Perceived Complexity:**

**Please rate your level of agreement with the following statements:**

Strongly Disagree | Disagree | Neutral | Agree | Strongly Agree

1. "How strongly do you agree with the following statements?

- Collaborating with academics is too complex and time-consuming.
- The academic research process is not compatible with our product development timeline.
- The regulatory and ethical requirements for academic trials are too burdensome.

**Perceived Value:**

1. "How strongly do you agree with the following statements?

- Academic trials would not add significant value to our intervention.
- Our internal evaluations are sufficient to validate our digital mental health intervention.
- The outcomes of academic research are not directly applicable to our business needs.

**Decision-Making Factors**

**Business Priorities:**

39. "How important are the following factors in your decision not to pursue academic collaborations? (Rate each on a scale of 1-5, where 1 = Not important and 5 = Extremely important)"

- Speed to market
- Maintaining control over the product development process
- Focus on user feedback rather than academic validation
- Avoiding potential delays associated with academic research

**Previous Experiences:**

40."If your organisation has previously considered or initiated a collaboration with academic researchers but did not follow through, what were the key reasons for not proceeding? (Select all that apply)"

- Lack of clear benefits
- Mismatch in goals or expectations
- Complexities in setting up the collaboration
- Budgetary constraints
- Concerns about intellectual property rights
- Other (please specify)
- N/A – not considered

**Open-Ended Questions**

**Key Concerns:**

41. "What are the main concerns or challenges your organisation has about collaborating with academic researchers?"

**Potential Motivators:**

42. "What would motivate your organisation to consider collaborating with academic researchers in the future?"

**Interest in Future Collaboration:**

43. "Would your organisation be open to exploring academic collaborations in the future if the barriers you identified were addressed?"

- Yes
- No
- Maybe

**Suggestions for Support:**

44. "What support or resources would help your organisation consider academic collaborations for evaluating or trialling your digital mental health intervention?"

**Appendix D - The 14 principles of engagement**

# UNITE: Principles of Engagement for Industry-Academic Partnerships in Mental Health Research

# Project Initiation

1. **Promote early collaborations**

**Action:** Industry, academics and Patient and Public Involvement (PPI) members should consider partnering early in the development cycle and engaging in more blue-sky discussions. This might be achieved via scheduled catch-ups to discuss on-going developments with key members of each team. Consider using an innovation workspace to support this. Both industry and academic partners should be prepared to sign non-disclosure agreements.

**Why**: To ensure that partners are up to speed and able to provide timely and informed support once it is time to apply for funding.

1. **Cost-Effective Funding Models:**

**Action**: It is important for universities to provide competitive pricing for research-based projects, to encourage collaboration and increase their portfolio of industry research. This might include developing cost-sharing models or partial funding arrangements that specifically cater to smaller businesses or startups. For instance, universities could partner with industry to jointly apply for funding, ensuring that smaller companies can participate without bearing the full financial burden.

**Action:** Universities need to be aware of how they present their costs in a way which does not alienate Industry partners. For example, consider providing a daily cost for each member of the team, which is inclusive of overheads costs.

**Action:** Universities should consider providing a short statement of work, specifying provision to wider access to resources (e.g., software) and depth of expertise and experience provided by individuals/team members.

**Why**: These actions will help support small and/or start-up businesses to engage with academics. Effective costing models reduces the barriers to collaboration and promote equality in access to academic expertise. Providing a single cost that does not separate overheads, it is more congruent with the pricing structures used by industry partners and consultants.

# Defining the Scope and Agreements

1. **Standardised Contract Templates:**

**Action**: Universities should work to create standardised contract templates for collaborations with industry. These should address common issues like IP rights, confidentiality, and funding arrangements to reduce contracting delays. Existing toolkits, such as the [Lambert Toolkit](https://www.gov.uk/guidance/university-and-business-collaboration-agreements-lambert-toolkit) could be used as a basis for developing collaboration agreements.

**Action**: Universities should explore having an organisation that can act as its own vehicle and facilitate collaborations and sign contracts up to a certain level. For example, this might include the option to offer a flexible pricing up to an initial review period (e.g. 12 months) to help facilitate early collaborations with industry. Within this model the day rates are agreed a priori by the university and thus procurement do not need to approve it. If taken further than the agreed initial period, this might then require further review through standard university processes.

**Why**: This approach allows academics to have autonomy to do this up to the point they have a contract – facilitating a proactive rather than reactive climate– the reactive element is slow. By reducing the back-and-forth over contracts, both industry and academia can initiate projects more quickly and efficiently.

1. **Streamlined IP Negotiations:**

**Action**: Establish clear guidelines and agreements for IP ownership and commercialisation at the outset of the project. This should be overseen by a Tech Transfer Office (or equivalent) within a university. Engaging legal experts who understand the unique needs of both sectors can help simplify this process.

**Why**: Reduces ambiguity and ensures that both parties are on the same page regarding ownership, licensing, and commercialisation rights. Having clear IP guidelines early in the project helps avoid delays at later stages.

1. **Role of a Liaison or Mediator:**

**Action**: Appoint a liaison or mediator who is familiar with both industry and academic cultures to help manage expectations and bridge any gaps in communication. This person should serve as a go-between when conflicts arise due to cultural differences in how work is done.

Harnessing relationships with external partners such as Health Innovation Networks or MindTech HRC is a useful way of bringing academia and industry together and brokering early relationships. Additionally, a Knowledge Transfer Partnership (KTP) fellow can be useful in bridging the gap between the two and acting as a mediator and translator.

**Why**: A liaison/mediator can ensure smoother communication and prevent misalignment by interpreting each side’s needs and helping to resolve misunderstandings in real-time.

1. **WOW (ways of working) Workshop:**

**Action**: Schedule a “Ways of Working” (WOW) workshop at the start of the project to introduce industry, academic and PPI partners to the work cultures and expectations on both sides. The workshop should aim to establish mutual understanding, align expectations, and build rapport between stakeholders. Industry partners value a flexible and organic collaboration style that encourages innovation, while academia should be open to non-traditional, less rigid structures that allow for greater creativity. PPI members value transparency, meaningful involvement, accessible/jargon-free communication and fair recognition.

**Action:** During this workshop, the teams should understand and document the risk appetite of each partner. This could be classified using a Red, Amber, Green (RAG) rating scale.

After understanding the appetite for risk, the team can then set out appropriate ways of working. For example, adopting a 'fast fail' approach for academic and industry collaborations may be beneficial, as it would allow teams to quickly identify and address potential issues, thus enabling them to pivot or refine their strategies early in the process. This would save time and resources while fostering innovation and knowledge.

**Action:** The details of this workshop should inform the joint project charter.

**Why**: Helps establish mutual respect, reduces potential frustration, and fosters a more collaborative environment by understanding the underlying cultural differences.

1. **Develop a Joint Project Charter**

**Action:** At the beginning of any collaboration, industry and academic partners, including PPI experts, should co-create a Project Charter that outlines the goals, timelines, deliverables, and expectations for both speed and rigor. This document should specify any areas where flexibility is allowed (e.g., adjusting timelines or methodology) and where rigor is non-negotiable (e.g., peer review, ethical standards). Open and early discussions about project timelines, key milestones, expectations, roles, and responsibilities are crucial for aligning both perspectives. Partners should be encouraged to review the Project Charter periodically. The charter could also outline steps to take when a disagreement or dispute between industry and academic partners arise.

The Chater should also outline principles of collaboration involving PPI, including what is reasonable and safe to ask for PPI members.

The Chater also provides a clear reference source and pathway for addressing issues that may arise, minimising delays and maintaining positive working relationships.

**Action:** Create a RACI matrix (Responsible, Accountable, Consulted, and Informed) for every collaborator involved in the project. This will clearly define who is responsible for what tasks, who needs to be consulted for input, and who is simply kept informed.

**Why:** Reduces ambiguity in decision-making and ensures that everyone knows their duties, leading to quicker progress and fewer delays. Ensures all parties have aligned expectations from the start and have a reference point for any necessary adjustments as the project progresses.

# Project Execution

1. **Dedicated Project Management Team:**

**Action:** Set up a dedicated project management team comprising members from both academia and industry who are responsible for daily operations and communications. This team should track progress, address challenges, and ensure both sides are meeting expectations. Industry partners value working with junior academics who may have more capacity to deliver on action points.

**Why:** A dedicated team can provide consistent oversight, identify potential challenges early, and keep the project moving forward without miscommunication.

1. **Adopt Appropriate Methodology Integration**

**Action**: Teams should be open to “thinking like a start-up, working like a university” which might embody new ways of working.

Teams should consider appropriate methodologies for ways of working at the project onset. For example, this might require adopting elements of agile project management where appropriate, especially for faster-paced elements of the research, such as data collection and initial analysis. Agile project management involves iterative cycles, sprints, and continuous feedback loops. Consider implementing Rapid Application Development (RAD) principles creating something quickly and efficiently. Ensure all team members are supportive of and informed of the methodology at project initiation.

**Why**: Promotes faster decision-making and adjustment, keeping momentum while ensuring that long-term findings are still rigorously validated by academia.

1. **Use a Shared Digital Workspace:**

**Action:** Utilise a shared digital platform (e.g., Google Drive, Microsoft Teams, Trello) where all documents, progress updates, and feedback are stored. This allows all parties to monitor progress in real-time and provides transparency.

**Why:** Ensures that everyone is on the same page, improves communication, and avoids duplicated work or misunderstandings.

1. **Meetings:**

**Action:** Schedule frequent follow-up meetings (e.g., bi-weekly or monthly) in addition to the initial kick-off meeting. These should be focused on progress updates, expectation realignment, and tangible action items to keep the momentum going.

*Meetings should be lean:* It is important to keep meetings short, snappy and engaging. This involves well thought through agendas and good chairing. Often only relevant members of the team who have the capacity to make direct actions are required at most meetings.

**Why:** Regular touchpoints keep the collaboration on track and ensure both sides are aligned in terms of deliverables, goals, and timelines. Efficient meetings support project progression as well as improve the quality of relationships.

1. **Celebrate Milestones and Successes:**

**Action:** At key stages of the collaboration (e.g., project completion, successful publication, or product launch), acknowledge achievements with joint celebrations or recognition. This fosters goodwill and motivates both sides to continue working together.

**Why:** Recognition of success creates positive momentum and strengthens the relationship between academia and industry, leading to future collaborations

# Promoting Sustainability

1. **Structured Internship or Fellowship Programs:**

**Action:** Implement formal internship or fellowship programs that allow postgraduate students or early-career researchers to spend time working with industry partners. These programs should be designed to expose students to practical, real-world challenges in mental health research. Consider exploring funding routes such as the [UK Research and Innovation (UKRI) Knowledge Transfer Partnership (KTPs)](https://www.ukri.org/councils/innovate-uk/guidance-for-applicants/guidance-for-specific-funds/knowledge-transfer-partnership-guidance/).

**Why:** Encourages talent development, ensures fresh perspectives, and strengthens the academic-industry pipeline**.**

1. **Mentoring and Career Development:**

**Action**: Create mentorship opportunities where senior academic researchers can mentor early-stage researchers working with industry partners (and vice versa), helping to bridge the gap.

**Why**: Supports talent retention and builds relationships that may lead to long-term collaboration.
